# A Clinical Prognostic Model Based on Machine Learning from the Fondazione Italiana Linfomi (FIL) MCL0208 Phase III Trial

**DOI:** 10.3390/cancers14010188

**Published:** 2021-12-31

**Authors:** Gian Maria Zaccaria, Simone Ferrero, Eva Hoster, Roberto Passera, Andrea Evangelista, Elisa Genuardi, Daniela Drandi, Marco Ghislieri, Daniela Barbero, Ilaria Del Giudice, Monica Tani, Riccardo Moia, Stefano Volpetti, Maria Giuseppina Cabras, Nicola Di Renzo, Francesco Merli, Daniele Vallisa, Michele Spina, Anna Pascarella, Giancarlo Latte, Caterina Patti, Alberto Fabbri, Attilio Guarini, Umberto Vitolo, Olivier Hermine, Hanneke C Kluin-Nelemans, Sergio Cortelazzo, Martin Dreyling, Marco Ladetto

**Affiliations:** 1 Unit of Hematology, Department of Molecular Biotechnology and Health Sciences, University of Torino, 10126 Torino, Italy; simone.ferrero@unito.it (S.F.); elisa.genuardi@unito.it (E.G.); daniela.drandi@unito.it (D.D.); daniela_barbero@alice.it (D.B.); 2 Unit of Hematology and Cell Therapy, IRCCS-Istituto Tumori ‘Giovanni Paolo II’, 70124 Bari, Italy; attilioguarini@oncologico.bari.it; 3 Institute of Medical Informatics, Biometry, and Epidemiology, Ludwig-Maximilians-University of Munich, 81377 Munich, Germany; Eva.Hoster@med.uni-muenchen.de; 4 Division of Nuclear Medicine, University of Torino, 10126 Turin, Italy; passera.roberto@gmail.com; 5 Unit of Clinical Epidemiology, CPO Piemonte, AOU Città della Salute e della Scienza di Torino, 10126 Turin, Italy; andrea.evangelista@cpo.it; 6 Department of Electronics and Telecommunications, Politecnico di Torino, 10129 Turin, Italy; marco.ghislieri@polito.it; 7 PoliToBIOMedLab of Politecnico di Torino, 10129 Turin, Italy; 8 Hematology, Department of Translational and Precision Medicine, Sapienza University of Rome, 00161 Rome, Italy; delgiudice@bce.uniroma1.it; 9 Hematology Unit, Santa Maria delle Croci Hospital, 48121 Ravenna, Italy; monica.tani@auslromagna.it; 10 Division of Hematology, Department of Translational Medicine, University of Eastern Piedmont, 28100 Novara, Italy; riccardo.moia@uniupo.it (R.M.); marco.ladetto@ospedale.al.it (M.L.); 11 Unit of Hematology, Presidio Ospedaliero Universitario “Santa Maria della Misericordia”, Azienda Sanitaria Universitaria Friuli Centrale, 33100 Udine, Italy; stefano.volpetti@asuiud.sanita.fvg.it; 12 Unit of Hematology and Bone Marrow Transplant, Businco Hospital, 09121 Cagliari, Italy; cabras.giuseppina@tiscali.it; 13 Unit of Hematology and Bone Marrow Transplant, ‘V. Fazzi’ Hospital, 73100 Lecce, Italy; direnzo.ematolecce@gmail.com; 14 Hematology, AUSL/IRCCS, 42123 Reggio Emilia, Italy; Francesco.Merli@ausl.re.it; 15 Unit of Hematology, Department of Oncology and Hematology, Guglielmo da Saliceto Hospital, 29121 Piacenza, Italy; d.vallisa@ausl.pc.it; 16 Division of Medical Oncology and Immune-Related Tumors, Centro di Riferimento Oncologico di Aviano (CRO) IRCCS, 33081 Aviano, Italy; mspina@cro.it; 17 Unit of Hematology, dell’ Angelo Mestre-Venezia Hospital, 30174 Mestre-Venezia, Italy; anna.pascarella@aulss3.veneto.it; 18 Unit of Hematology and Bone Marrow Transplant, ‘San Francesco’ Hospital, 08100 Nuoro, Italy; gclatte@gmail.com; 19 Unit of Hematology, Azienda Ospedali Riuniti Villa Sofia-Cervello, 90146 Palermo, Italy; k.patti@villasofia.it; 20 Unit of Hematology, Azienda Ospedaliera Universitaria Senese, 53100 Siena, Italy; fabbri7@unisi.it; 21 Division of Hematology, Azienda Ospedaliero Universitaria Città della Salute e della Scienza di Torino, 10126 Turin, Italy; umberto.vitolo@ircc.it; 22 Service D’hématologie, Hôpital Universitaire Necker, Université René Descartes, Assistance Publique Hôpitaux de Paris, 75015 Paris, France; ohermine@gmail.com; 23 Department of Haematology, University Medical Center Groningen, University of Groningen, 9713 Groningen, The Netherlands; j.c.kluin@umcg.nl; 24 Unit of Oncology, Humanitas/Gavazzeni Clinic, 24125 Bergamo, Italy; sergio.cortelazzo@gmail.com; 25 Department of Medicine III, University Hospital, LMU Munich, 81377 Munich, Germany; Martin.Dreyling@med.uni-muenchen.de; 26 Division of Hematology, Azienda Ospedaliera SS Antonio e Biagio e Cesare Arrigo, 15121 Alessandria, Italy

**Keywords:** machine-learning, mantle cell lymphoma, prognostication

## Abstract

**Simple Summary:**

The interest in using Machine-Learning (ML) techniques in clinical research is growing. We applied ML to build up a novel prognostic model from patients affected with Mantle Cell Lymphoma (MCL) enrolled in a phase III open-labeled, randomized clinical trial from the Fondazione Italiana Linfomi (FIL)—MCL0208. This is the first application of ML in a prospective clinical trial on MCL lymphoma. We applied a novel ML pipeline to a large cohort of patients for which several clinical variables have been collected at baseline, and assessed their prognostic value based on overall survival. We validated it on two independent data series provided by European MCL Network. Due to its flexibility, we believe that ML would be of tremendous help in the development of a novel MCL prognostic score aimed at re-defining risk stratification.

**Abstract:**

Background: Multicenter clinical trials are producing growing amounts of clinical data. Machine Learning (ML) might facilitate the discovery of novel tools for prognostication and disease-stratification. Taking advantage of a systematic collection of multiple variables, we developed a model derived from data collected on 300 patients with mantle cell lymphoma (MCL) from the Fondazione Italiana Linfomi-MCL0208 phase III trial (NCT02354313). Methods: We developed a score with a clustering algorithm applied to clinical variables. The candidate score was correlated to overall survival (OS) and validated in two independent data series from the European MCL Network (NCT00209222, NCT00209209); Results: Three groups of patients were significantly discriminated: Low, Intermediate (Int), and High risk (High). Seven discriminants were identified by a feature reduction approach: albumin, Ki-67, lactate dehydrogenase, lymphocytes, platelets, bone marrow infiltration, and B-symptoms. Accordingly, patients in the Int and High groups had shorter OS rates than those in the Low and Int groups, respectively (Int→Low, HR: 3.1, 95% CI: 1.0–9.6; High→Int, HR: 2.3, 95% CI: 1.5–4.7). Based on the 7 markers, we defined the engineered MCL international prognostic index (eMIPI), which was validated and confirmed in two independent cohorts; Conclusions: We developed and validated a ML-based prognostic model for MCL. Even when currently limited to baseline predictors, our approach has high scalability potential.

## 1. Introduction

Currently, prospective multicenter clinical trials are accumulating unprecedented amounts of information. The potential of these data is underexploited, in terms of increasing our understanding of the diseases and our ability to discriminate their outcomes [1].

Although in its infancy, the application of machine-learning (ML) tools in oncology and hematology is currently on the rise [2,3]. In acute myeloid leukemia, ML has been applied to drug discovery programs and gene expression profiling, leading to the discovery of novel predictive biomarkers [4,5,6]. Moreover, ML can be applied to the development of prediction models of treatment–response optimal timing [7,8], hematopoietic stem cell transplantation outcomes [9,10,11,12], and survival outcomes [13,14,15,16]. For example, Biccler et al. exploited registry data to develop several prognostic models for diffuse large B-cell lymphoma (DLBCL) [13]. Their ML approach identified clinical prognostic factors that performed better than the International Prognostic Index (IPI), in training-set and validation-set, respectively.

Mantle cell lymphoma (MCL) is a highly heterogeneous disease. Some subtypes are aggressive and chemo-refractory; however, other subtypes have shown prolonged survival after tailored treatment [17,18,19,20]. Currently, a number of prognostic models are available that are generally related to the MCL international prognostic index (MIPI) [21,22,23,24,25]. The standard MIPI (MIPI-st) was developed by Hoster et al., and it has been refined and adapted over time.

Taking advantage of our experience with the MCL0208 clinical trial for young patients with MCL [26] (NCT02354313, sponsored by the Fondazione Italiana Linfomi [FIL]), we systematically collected and organized hundreds of clinical and biological variables in a previously generated data warehouse (DW) [1,27,28], which allowing careful quality assessments and substantial improvements in the accuracy of the results [29].

In the present study, we applied a hierarchical clustering algorithm to a large number of clinical variables from the DW, collected at baseline. We assessed their prognostic value on overall survival (OS) and, following the clustering analysis, we modeled a novel prognostic score, which we defined as the engineered MIPI (eMIPI). This was finally validated in two independent data series from the European MCL Network (NCT00209222, NCT00209209).

## 2. Materials and Methods

### 2.1. Patients

Data were collected from a phase III, multicenter, open-label, randomized, controlled clinical trial, primarily aimed at determining the efficacy and safety of Lenalidomide as a 2 years maintenance therapy after autologous stem cell transplantation (ASCT). The trial enrolled 303 younger (≤65 years) patients with MCL, all of which received high-dose immune-chemotherapy, followed by ASCT [26]. The study was conducted in accordance with the Declaration of Helsinki, and all patients provided written informed consent for the collection and research use of clinical and biological data.

### 2.2. Data Preparation

Data preparation is described in the Supplementary Methods and Figure S1. We retrieved 34 available clinical features at baseline from electronic case report forms and laboratory data sources. These features included clinical (e.g., Eastern Cooperative Oncology Group parameters), laboratory (e.g., lactate dehydrogenase [LDH] below or above the upper limit of normal [ULN]), pathology (e.g., Ki-67 proliferation index), and demographic (age at diagnosis) variables.

Among these 34 features, 8 were not eligible for analysis, due to the high number of missing values, and were thus excluded. Among the remaining others, 17 were continuous and 9 were binary: the continuous variables were dichotomized according to established cut-offs to allow comparisons:

- 14 features dichotomized assuming the abnormal vs. normal range according to the literature [30] (see Supplementary Methods).
- Ki-67 was categorized according to the recognized cut-off (≥30%) from the literature [31].
- Regarding the Age at diagnosis and the lymphoma involvement by flow-cytometry on peripheral blood (flowPB) variables, an optimal cut-off was respectively determined by applying a spline function fitted via logistic regression model, assuming the PFS at June 2019 data cut-off as a dependent variable.

Only patients without missing values were included in the training-set.

### 2.3. Clustering Analysis and Features Reduction

Clustering analysis was performed on complete data to discriminate different groups of patients, based on their baseline features (Figure S2). We applied a hierarchical algorithm setting the “Ward” linkage and the “Euclidean” distance. The cluster analysis was implemented via Matlab R2019 (version 9.8.0.1359463 (2020a), Natick MA, USA, Bioinformatics Toolbox.

The acquired groups of patients were then correlated with clinical outcomes, and the best model was assessed with a metric to allow comparison between survival models, including concordance (C)-index [32], -2*log-likelihood (-2LL), Akaike (AIC), and Bayesian (BIC) Information Criteria calculations. The best model was then chosen for further analytical steps.

To select a clinically applicable set of variables, we firstly applied a statistical bivariate feature reduction (as detailed in the Supplementary Methods). For the ultimate feature selection, we applied a Recursive Feature Extraction algorithm (RFE, Figure S2F) with the caret package (V. 6.0-84), provided with R-Project software (version 1.2.5042, R Core Team [2020], Vienna, Austria, https://www.r-project.org). A resampling method was applied as cross-validation. The training set was randomly divided into 10 parts and then each part was used as testing dataset for a Random Forest model trained on the other 9 (10-fold cross-validation). The accuracy given by each model was assessed by calculating the average of 5 error terms obtained by performing 10 folds five times. Based on the most accurate model, we selected the number of the most influencing features, and of these bases we defined the eMIPI score (Figures S2G and S5).

### 2.4. Survival Analysis

Survival analyses were performed with the training-set, according to eMIPI classes, with both multivariate Cox and Kaplan-Meier (K-M) methods (survival data cut-off: June 2019). Then, the eMIPI classifications were compared to previously recognized prognostic models: the MIPI-st, according to Hoster et al. [21], the MIPI-biological (b) [21], and the MIPI-c [22] (Figure S2H). The models were compared by assessing C-index, -2LL, AIC, and BIC. The outcome analysis, Cox modeling, and performance of each model were implemented with the “Survival” (V. 2.44-1.1), and “stats” (V 3.6.2.) packages provided with R. To validate our methods, we used the Transparent Reporting of a Multivariable Prediction Model for Individual Prognosis OR Diagnosis (TRIPOD) criteria.

### 2.5. Extrapolation of a Simplified eMIPI Score for External Validation

Reproducible formulas were implemented to assign patients to eMIPI prognostic groups (Figure S2I), according to each patient’s profile. The total set of patient profiles was thus extracted from a heat-map, where the classification was assigned according to the outcome.

Next, we externally validated the eMIPI on a trial cohort of “Younger” patients from the European MCL Network, that was comparable to the FIL-MCL0208 discovery cohort (Figure S2J). We also validated the eMIPI on a trial cohort of “Elderly” patients from the European MCL Network and we explored the prognostic value of the eMIPI for clinical outcomes by comparing it to the prognostic values of the MIPI-st, MIPI-b, and MIPI-c. Validation methods are detailed in the Supplementary methods.

## 3. Results

### 3.1. Patient Characteristics

Demographic and clinical characteristics from the 300 patients eligible are summarized in Table 1 [26].

Overall, 185 patients were considered for the training-set. For OS, the median follow-up was 4.7 years, with an interquartile range (IQR) of 4.3–5.2 years. For progression-free survival (PFS), the median follow-up was 4.8 years (IQR: 4.3–5.3), and the five-year PFS was 52%. OS probability of patients included vs. excluded (N = 115) from the training-set were superimposable, as shown in Figure S3.

According to the MIPI-st, we classified 110 (59%) patients as Low risk, 53 (27%) patients as intermediate risk (Int), and 22 (12%) patients as High risk. According to the MIPI-b, we classified 49 (26%) patients as Low risk, 87 (47%) patients as Int risk, and 49 (26%) patients as High risk. Finally, according to the MIPI-c, we classified 91 (49%) patients as Low risk, 49 (26%) patients as Int-Low risk, 28 (15%) patients as Int-High risk, and 17 (10%) patients as High risk.

### 3.2. Clustering Analysis from the Whole Set of Features

**Figure 1:**
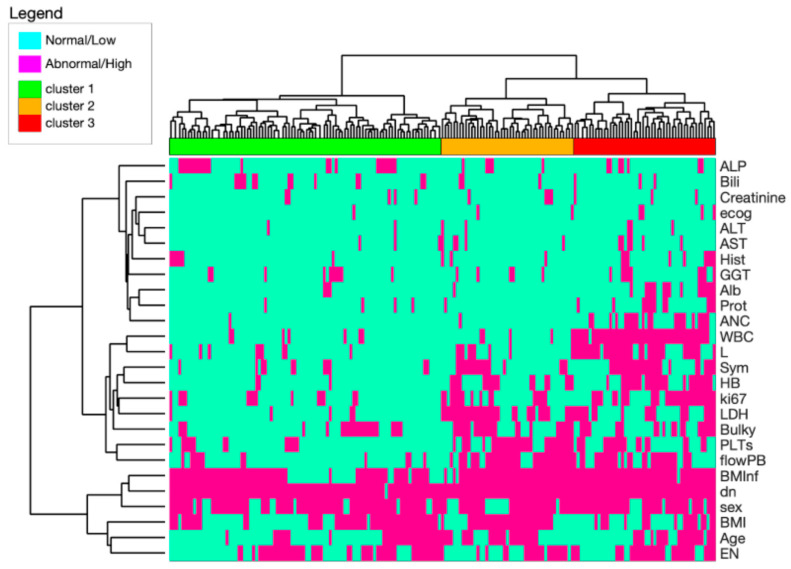
Heat-map of potential prognostic factors for MCL, based on a cluster analysis on training dataset. Light green cells represent either normal or low values of the indicated variables; magenta cells represent either abnormal or high values of the indicated variables. Three large clusters emerged that were associated with Low (green, C1), Intermediate (orange, C2), and High (red, C3) risk. Abbreviations. ALP: alkaline phosphatase; Bili: bilirubin, ecog: performance status; ALT: alanine aminotransferase; ASP: aspartate aminotransferase; Hist: histology; GGT: gamma glutamyl-transferase; Alb: albumins; Prot: total proteins; ANC: absolute neutrophil count; WBC: white blood cell count; L: lymphocytes; Sym: B symptoms; HB: hemoglobin; ki67: cell proliferation marker; LDH: lactate dehydrogenase; PLTs; platelets; flowPB: lymphoma involvement, revealed with flow-cytometry analysis of peripheral blood; BMInf: tumor infiltration, assessed with immunohistochemistry on bone marrow biopsies; dn: nodal involvement, based on a computerized tomography scan; BMI: body mass index; EN: extranodal involvement, based on computerized tomography scan.

Figure 1 shows the heat-map that was constructed based on the clustering analysis of the training-set. The horizontal dendrogram is the result of patients clustering, while the vertical dendrogram outlines the clustering of patient characteristics. This analysis allowed us to define three clusters (C) of patients, designated as: C1 (*n =* 92, 50%), C2 (*n =* 45, 24%), and C3 (*n =* 48, 26%). A correlation analysis between each group and the clinical outcomes indicated that the OS model outperformed the PFS model (C-index: 0.64, standard error [se] = 0.03 vs. 0.60, se = 0.03; -2LL: 392.1 vs. 798.2; AIC: 398.1 vs. 802.2; and BIC: 401.5 vs. 807.1). Thus, the OS model was selected for further analyses.

### 3.3. Clustering on the Clinically Relevant Variables: The eMIPI Definition

#### 3.3.1. Feature Reduction

The final model selection fulfilled the clinical requirement for obtaining a signature of a few clinical variables that were easily derived from patient characteristics (Supplementary Results). The final model was tested on patients that were classified based on three groups with significantly different risks of OS. The signature selected by the best performing model included the following seven predictors: albumin levels; Ki-67 staining; LDH below or above the ULN; lymphocytes (L); platelet levels; tumor infiltration assessed by morphology and immunohistochemistry on bone marrow biopsy (BMInf); and B-symptoms.

**Figure 2:**
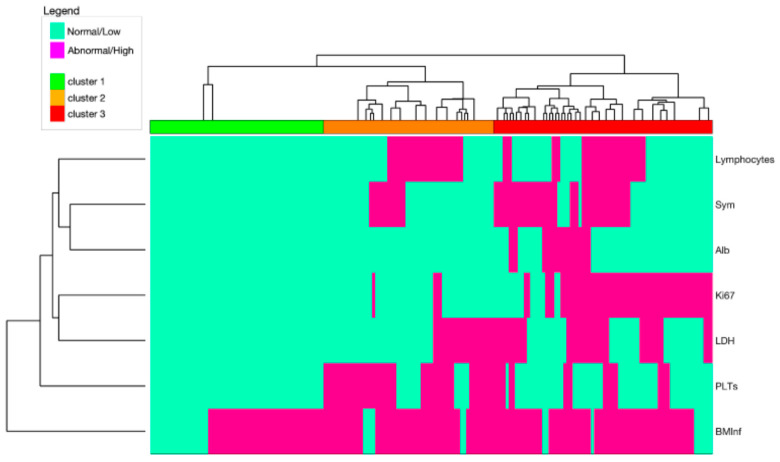
Heat-map of seven selected potential prognostic factors for MCL, based on a cluster analysis on training dataset. Light Green cells represent either normal or low values of the indicated variables; magenta cells represent either abnormal or high values of the indicated variables. Three large clusters emerged that were associated with Low (green), Intermediate (orange), and High (red) risk. Abbreviations. Sym: B symptoms; Alb: albumin; Ki67: cell proliferation marker; LDH: lactate dehydrogenase; PLTs: platelets; BMInf: tumor infiltration, assessed with immunohistochemistry on a bone marrow biopsy.

The same clustering procedure was then repeated, this time involving only these 7 aforementioned variables (Figure 2). Additionally, in this case, the heatmap showed three different clusters of patients: C1 (*n =* 57, 31%), C2 (*n =* 56, 30%), and C3 (*n =* 72, 39%). As in the starting model, we correlated each group with clinical outcomes and observed that the OS-based model outperformed the model based on the PFS (C-index: 0.69, se = 0.04 vs. 0.63, se = 0.03; -2LL: 381.9 vs. 791.2; AIC: 383.3 vs. 795.2; and BIC: 385.6 vs. 800.1).

#### 3.3.2. Comparison between the Simplified and Starting Models

We compared the starting model, which included all 26 features (Figure 1), to the simplified model composed of only seven features (Figure 2). The latter model slightly outperformed the starting model, including the whole set of variables in predicting OS (C-index: 0.69, se = 0.04 vs. 0.64, se = 0.03; -2LL: 381.9 vs. 392.1; AIC: 383.9 vs. 398.1; and BIC: 385.6 vs. 401.5).

#### 3.3.3. Survival Analysis

**Figure 3:**
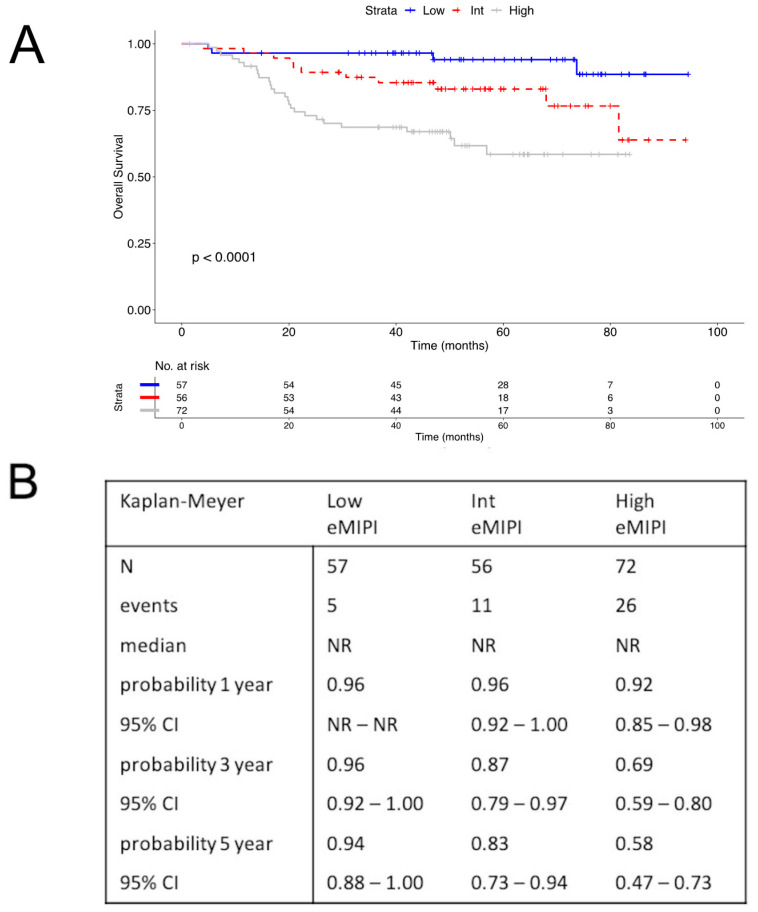
Survival among patients with MCL, according to eMIPI values. (**A**) Kaplan-Meier curve shows OS and numbers at risk for patients with Low, Int, and High eMIPI values. (**B**) OS estimated at 1, 3, and 5 years, in patients with Low, Int, and High eMIPI values. Abbreviations: MCL: mantle cell lymphoma; MIPI: international MCL prognostic index; eMIPI: engineered MIPI; OS: overall survival; N: number; Int: intermediate; NR: not reached; CI: confidence interval.

With the simplified model, we prepared K-M survival curves with patients stratified according to the C1, C2, and C3 patient groups. This analysis showed that the three groups had significantly different risk of OS. Hence, these risk groups were renamed in terms of the eMIPI, as Low, Int, and High, respectively. Figure 3A shows the K-M curves of OS for the three eMIPI groups. The cumulative survival probabilities at 5 y were 0.94, 0.83, and 0.58, for the Low, Int, and High eMIPI groups, respectively (Figure 3B). We observed that patients High eMIPI values had a significantly lower OS than those with Int (HR: 2.32, 95% CI: 1.14–4.73, *p* = 0.025) and Low eMIPI values (HR: 7.09, 95% CI: 2.46–20.48, *p* < 0.001).

### 3.4. Patient Profiles According to eMIPI

To create a simple prognostic tool for validation on an external cohort series, we analyzed each patient profile obtained from the cluster analysis (a total of fifty-five possible profiles), representing every eMIPI class (Table S2). The simplification rules derived from these profiles are shown in Table 2.

Most patient profiles could be readily assigned to the three main groups with Low, High, and Int. In some cases, those patient profiles that could not be assigned to either the Low risk or the High risk groups were assigned to the Int risk group (Table 2, formula 8).

Briefly, patients with abnormal albumin were always classified as High risk, according to the heatmap. Additionally, some patients with normal albumin were characterized as High risk on the basis of abnormal values for the other remaining features (Table 2, formulas 3–7). 

Notably, we individually tested each simplified formula by comparing the resulting eMIPI class of risk with clinical outcomes to verify the correctness of each formula. A K-M survival analysis confirmed that the formulas provided consistent classifications, as expected from the Figure 3A.

### 3.5. eMIPI Comparison with Recnognized Scores

We compared the eMIPI classification with three currently recognized indexes for predicting the OS: the MIPI-st, the MIPI-b, and the MIPI-c. All indexes were tested on the same subset of patients.

**Figure 4:**
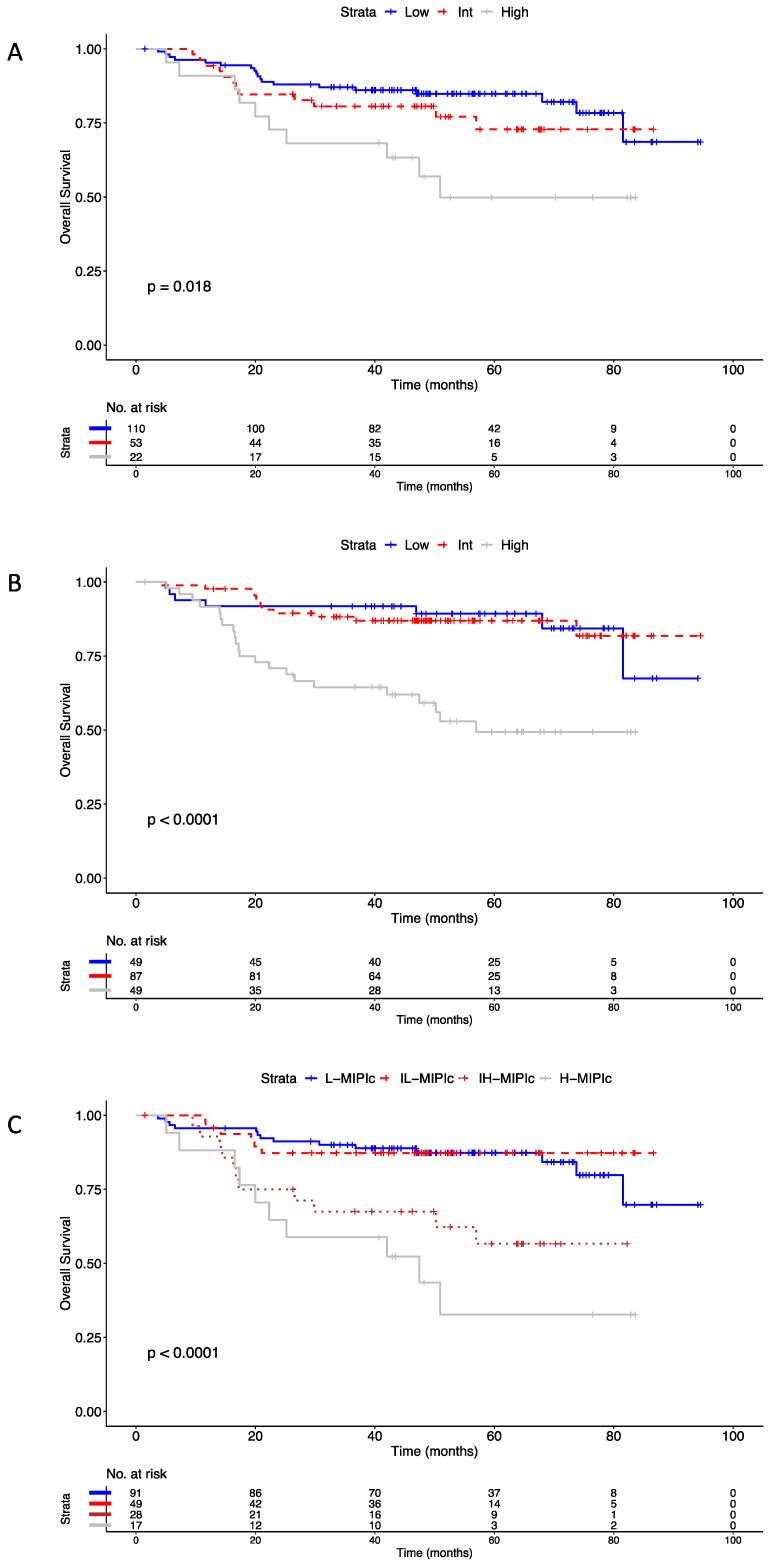
K-M survival plots of patients with MCL, from training-set, separated according to known prognostic scores. (**A**) The MIPI-st classified 110 patients as Low risk, 53 patients as Int risk, and 22 patients as High risk. (**B**) The MIPI-b classified 49 patients as Low risk, 87 patients as Int risk, and 49 patients as High risk. (**C**) The MIPI-c classified 91 patients as Low risk, 49 patients as Int Low risk, 28 patients as Int High risk, and 17 patients as High risk. Abbreviations. OS: overall survival; K-M: Kaplan-Meyer; MCL: mantle cell lymphoma; MIPI: international MCL prognostic index; MIPI-st: MIPI-standard; MIPI-b: MIPI-biologic; N: number; Int: intermediate; CI: confidence interval; HR: hazard ratio.

Based on OS, patients in the High group displayed a significantly worse prognosis compared to the Low risk patients (HR: 2.92, 95% CI: 1.35–6.29, *p* = 0.014) when classified with the MIPI-st (Figure 4A). A similar trend was also observed when comparing the High risk patients with both the Low (HR: 4.09, 95% CI: 1.74–9.61, *p* < 0.001) and Int (HR: 3.93, 95% CI: 1.94–7.96, *p* < 0.001) risk groups, when applying the MIPI-b classifier (Figure 4B). According to MIPI-c (Figure 4C), both High-Int and High risk groups had significantly different scores when compared to Low risk (High-Int, HR: 3.12, 95% CI: 1.41–6.88, *p* < 0.001; High, HR: 4.83, 95% CI: 2.14–10.92, *p* < 0.001) groups, respectively. The comparison of eMIPI to the MIPI-st, MIPI-b, and MIPI-c, based on OS, confirmed a superimposed prognostic value. In fact, the C-indexes were 0.69, se = 0.04 for eMIPI vs. 0.61, se = 0.04 for MIPI-st, 0.67, se = 0.04 for MIPI-b, and 0.66, se = 0.05 for MIPI-c. Consistently, -2LL, AIC, and BIC were 381.9, 383.9, and 385.6 for eMIPI vs. 395.2, 399.0, and 402.6 for MIPI-st, 382.2, 387.2, and 390.6 for MIPI-b, and 382.9, 388.9, and 394.0 for MIPI-c, respectively.

Interestingly, 27 (25%) patients classified as Low risk and 29 (55%) patients classified as Int risk with the MIPI-st were reclassified as High risk with the eMIPI (Table 3). According to the MIPI-st, 110 patients were classified as Low risk and among these patients the eMIPI classified 34 and 27 patients as Int and High risk, respectively. Similarly, the MIPI-b classifier categorized 49 and 87 patients as Low and Int risk, respectively. However, with the eMIPI, 57 and 56 patients were classified as Low and Int risk, respectively.

Taken together, the eMIPI produces the most balanced groups of patients (Low risk: 31%, Int risk: 30%, and High risk: 39%), when compared to the distributions produced with the MIPI-st (Low: 59%, Int: 29%, and High: 17%) and the MIPI-b (Low: 26%, Int: 62%, and High: 10%).

### 3.6. External Validation

We next sought to validate the eMIPI approach by applying it to the external patient series from the “Younger” and “Elderly” trials of the European MCL Network [18,33]. For the “Younger” cohort, 254 out of 613 patients were selected for the comparative analysis. Of note, the excluded patients did not display any significant difference in terms of median survival (10.0 vs. 11.0 years) (Figure S6). In contrast, a significant difference in terms of median survival (9.1 and 6.9 years) was observed when comparing selected vs excluded patients when pooling together the “Younger” and “Elderly” series (Figure S7). Again, no difference was observed when comparing the excluded patients from both the “Younger” and “Elderly” series (59% vs. 60%).

**Figure 5:**
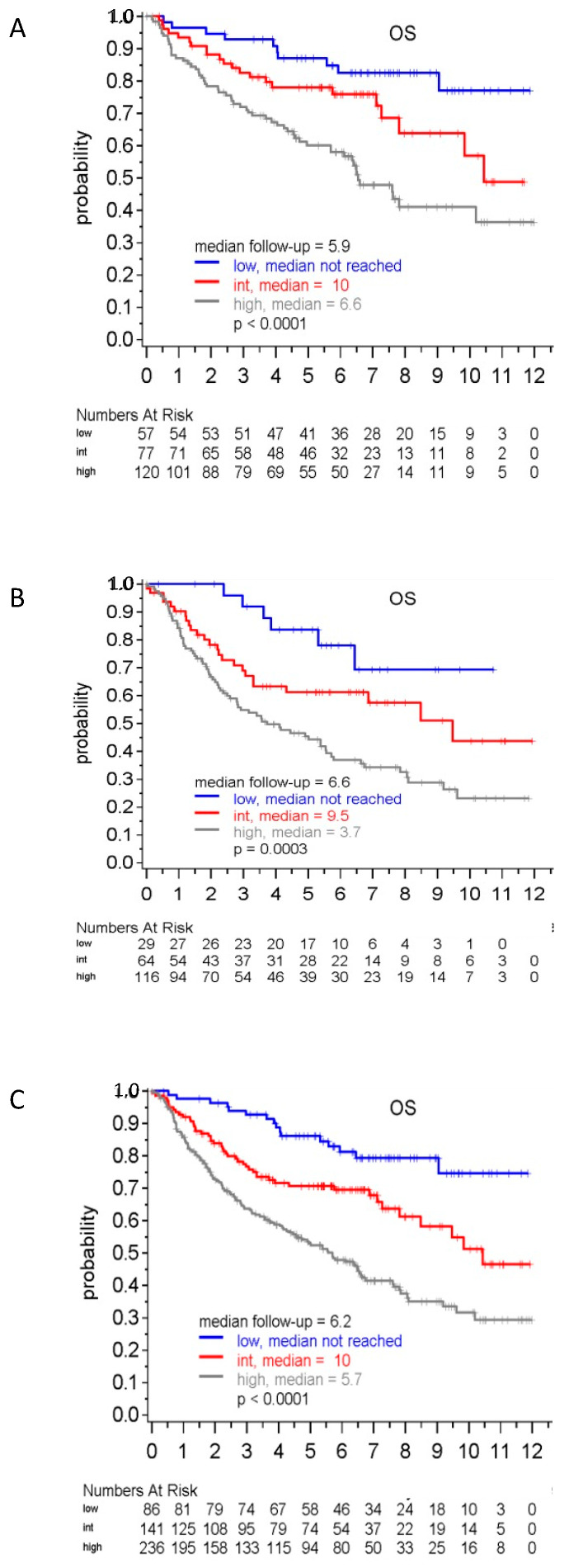
Prognostic value of eMIPI tested in the validation cohorts. (**A**) The validation cohort included pooled data from “Younger” individuals with MCL. (**B**) The validation cohort included pooled data from “Elderly” individuals with MCL. (**C**) The validation cohort included pooled data from “Younger” and “Elderly” individuals with MCL. Overall survival curves for the three risk groups and regression model analyses show distinctions between the different risk groups. Abbreviations. OS: overall survival; HR: hazard ratio; CI: confidence interval; Int: intermediate; MCL: mantle cell lymphoma; eMIPI: engineered international MCL prognostic index.

As per the prognostic value, the eMIPI discriminated three groups of patients from the “Younger” cohort: eMIPI Low (*n =* 86, 19%), eMIPI Int (*n =* 141, 30%), and eMIPI High (*n =* 236, 51%) (Figure 5A). In this series, patients from the eMIPI High group showed a significantly lower OS compared to the eMIPI Int (HR: 1.90, 95% CI: 1.30–2.60) and eMIPI Low groups (HR: 2.20, 95% CI: 1.20–3.40), respectively. In this validation-set, the eMIPI retained its prognostic value in reference to the recognized scores. The C-indexes for eMIPI vs. MIPI-st, MIPI-b, and MIPI-c, were: 0.63 vs. 0.63, 0.67, and 0.67, respectively. Consistently, -2LL, AIC, and BIC were 877.8, 883.8, and 888.8 for eMIPI vs. 877.8, 881.8, and 886.8 for MIPI-st, 856.8, 860.7, and 865.7 for MIPI-b, and 861.9, 867.9, and 875.3 for MIPI-c, respectively.

When surveying the prognostic value in the “Elderly” cohort (Figure 5B), the eMIPI-discriminated groups were composed of 57 (eMIPI Low, 22%), 77 (eMIPI Int, 29%), and 129 (eMIPI High, 49%) patients. Similarly, also in this cohort eMIPI High patients significantly displayed OS that the patients from both Int (HR: 1.90, 95% CI: 1.17–3.10) and Low (HR: 2.0, 95% CI: 0.95–4.20) groups. Additionally in this validation-series, the eMIPI retained its prognostic value in reference to the recognized scores with the C-index for eMIPI vs. MIPI-st, MIPI-b, and MIPI-c, being 0.61 vs. 0.62 and 0.63, and 0.66, respectively. Consistently, -2LL, AIC and BIC were 964.5, 968.5, and 973.8 for eMIPI vs. 962.1.8, 966.1, and 971.3 for MIPI-st, 952.4, 954.4, and 957.1 for MIPI-b, and 946.4, 952.4, and 960.3 for MIPI-c, respectively.

When pooling together the “Younger” and the “Elderly” series (Figure 5C), we observed patients with eMIPI High having a lower OS compared to eMIPI Int (HR: 1.80, 95% CI: 1.12–2.80) and Low eMIPI ones (HR: 2.20, 95% CI: 0.92–5.50). Consequently, the eMIPI retained its prognostic value in reference to the recognized scores also in this series (data not shown).

## 4. Discussion

In this study we developed a ML-based prognostic model to create a new MCL risk score, named eMIPI. The ML modeling approach included (i) clustering analysis using classical dendrograms and (ii) features reduction using a Random Forrest algorithm applied to a training cohort encompassing 300 patients (FIL-MCL0208). Finally, the robustness of our prognostic model was further validated using data from two large independent trials [18,33].

The application of ML approaches in the hematology field is rapidly growing, although most ML studies are retrospective [7,11,13,14,34,35,36], based on data retrieved from electronic health records at either single centers or multiple centers. For example, Agius et al. developed a ML pipeline based on data for 4149 patients retrieved from the Danish Chronic Lymphocytic Leukemia (CLL) registry. Those data allowed the construction of a very accurate treatment–infection model of CLL [35].

Clinical trials rarely allow researchers to collect the number of patients typically analyzed in retrospective series. However, trials often contain larger sets of variables and offer superior data quality, compared to those available for retrospective series. These observations were particularly evident in the FIL-MCL0208 trial, which underwent rigorous refinement, accurate feature assessments, and uniform evaluations of clinical outcomes through the DW-based data handling method [1]. Therefore, although the model proposed here did not take into account the full panel of data available from the eCRFs, it should be considered a first step in implementing reliable ML algorithms [37] in the context of a clinical trial.

Starting with thirteen baseline variables retrieved from a national registry, Biccler et al. showed that ML was useful in finding the most predictive model of risk among twelve supervised models for newly diagnosed DLBCL patients treated with rituximab, cyclophosphamide, doxorubicin, vincristine, and prednisolone (R-CHOP) or R-CHOP like therapy [8]. In our analysis, we started with thirty-four variables (Supplementary Figure S2A) as input for an unsupervised algorithm. Thus, data variability, when correctly handled, can allow the development of novel prognostic scores. Indeed, Kurtz et al. showed that a model that combined clinical data (IPI index), interim imaging risk factors, and circulating tumor DNA risk factors, outperformed each factor taken individually for predicting event-free survival among patients with DLBCL [38].

Overall, in this analysis a proportion of patients (38%) was excluded from the training set, due to the high number of missing values (Figure S1). This step was needed for clustering analysis, which runs only with complete data. Nonetheless, no selection bias was introduced in the analysis as the clinical outcome of included vs excluded patients was superimposable (Figure S3). On the other hand, we applied an unsupervised methodology which ensembled together several variables from different sources. At allowing a comparison with binarized variables, each continuous variable was iteratively dichotomized according to either recognized ranges or clinical outcomes (e.g., age at diagnosis and flowPB variables).

The FIL-MCL0208 DW contained a large number of variables. We chose to limit this first modeling effort to a subset of only 26 easily accessible variables for two reasons: (1) we needed to validate the model with an independent series that did not include all the biological features measured in the training-set; and (2) prognostic scores based on clinical variables easily accessible can provide greater opportunities, due to their broad applicability. However, we believe that models with more complex datasets will be feasible soon. Those studies will increase our knowledge of MCL biology and allow clinicians to choose the most robust biological predictors tailored to each case.

Differently from the recognized prognostic scores for MCL, the eMIPI included albumin levels (that might reflect the inflammatory status and the hepatic synthesis at diagnosis), B symptoms (included in the basic diagnostic workup for MCL), and BM tumor infiltration and altered PLTs levels (both possibly related to high tumor burden). Interestingly, abnormal levels of albumin are enough for conferring the patient to High risk profile.

Moreover, in both training and validation series, the eMIPI allocated a larger proportion of patients as High risk than recognized scores for patients of comparable age. This finding was critical, considering that MCL is still a frequently relapsing disease, and future trials that aim to test personalized treatment intensifications will benefit from prognosticators that can identify a considerable proportion of patients at High risk. To broadly promote the clinical usefulness of the eMIPI tool we implemented an easy-to-use calculator on the FIL website (http://filinf.it/eMIPI, accessed on 29 October 2021).

A partial drawback of this study is that the eMIPI did not outperform MIPI-st and MIPI-b when pooling together “Younger” and “Elderly” patients from European MCL Network. However, although the eMIPI was based on a cohort of young patients with MCL, it retained its prognostic value in a large trial of older patients. Thus, our results indicate that the variables chosen in our model are likely to retain good predictivity, regardless of the potential confounding roles of age- and frailty-associated parameters.

## 5. Conclusions

This study provided a proof-of-principle that ML can be a useful tool in prognostication modeling associated with clinical trials in lymphoma. We are aware that the eMIPI might potentially be integrated with biological and time-dependent variables in the future.

To fully exploit the potential of ML-based modeling, data might be pooled from several clinical trials with similar characteristics, and additional variables could be included. Application of the same principles to other disease entities might also be feasible.

## Figures and Tables

**Table 1:** Patient characteristics in the training-set (*n* = 185) compared to those excluded.

Patient Characteristics	Training Set, N (%) or Median (IQR), (MV)	Excluded Patients, N (%) or Median (IQR), (MV)	Cut-Off Values for Abnormal
Patients	185	115	-
Males	149 (81), (0)	86 (75), (0)	-
Age, y	57 (53, 62), (0)	56 (49, 60), (0)	-
Age ≥ 60, y *	73 (40)	-	-
BMI, kg/m^2^	26 (22, 28), (0)	25 (22, 27), (0)	-
BMI ≥ 25, kg/m^2^	99 (54), (0)	-	-
ECOGps ≥ 2	7 (4), (0)	3 (3), (0)	-
Sym	52 (28), (0)	31 (27), (0)	-
Bulky disease ≥ 5 cm	65 (35), (0)	33 (29), (0)	-
LDH ≥ upper limit of normal, UI/L	62 (34), (0)	47 (37), (0)	-
Platelets, 109/L	186 (133, 247), (0)	190 (133, 235), (0)	-
Platelets abnormal	61 (33)	-	<150 or >450
White blood cell count, 109/L	7 (6, 11), (0)	8 (6, 13), (0)	-
White blood cell counts abnormal	57 (31)	-	< 4 or > 11
Lymphocytes, 109/L	2 (1, 4), (0)	2 (2, 7), (0)	-
Lymphocytes abnormal	52 (28)	-	<1 or >5
ANC, 109/L	4 (3, 6), (0)	4 (3, 5), (0)	-
ANC abnormal	23 (12)	-	<1.5 or >8.0
Hb, g/dL	13 (12, 14), (0)	13 (11, 15), (0)	-
Hb abnormal	45 (24)	-	<11.7 or >18.0
ALT, IU/L	19 (13, 28), (0)	18 (14, 27), (0)	-
ALT abnormal	7 (4)	-	<7 or >56
AST, IU/L	20 (16, 26), (0)	20 (16, 26), (0)	-
AST abnormal	14 (8)	-	<10 or >40
Creatinine, mg/dL	0.9 (0.7, 1.0), (0)	0.9 (0.8, 1.0), (0)	-
Creatinine abnormal	12 (7)	-	Males: <0.5 or >1.2 Females: <0.4 or >1.1
Total Protein, g/dL	7.0 (6.7, 7.5), (0)	6.9 (6.6, 7.3), (16)	-
Total Protein abnormal	17 (9)	-	<6.0 or >8.3
Albumin, g/dL	4.1 (3.7, 4.4), (0)	4.2 (3.8–4.4), (31)	-
Albumin abnormal	19 (10)	-	<3.4 or >5.4
Bilirubin, mg/dL	0.5 (0.4, 0.7), (0)	0.5 (0.4–0.8), (17)	-
Bilirubin abnormal	20 (11)	-	<0.2 or >1.2
GGT, IU/L	26 (18, 40), (0)	25 (18–36), (17)	-
GGT abnormal	24 (13)	-	<8 or >65
ALP, IU/L	75 (58, 103), (0)	73 (59–102), (23)	-
ALP abnormal	34 (19)	-	<44 or >147
Ki-67, %	20 (10, 30), (0)	20 (10, 30), (25)	-
Ki-67 ≥ 30%	59 (32)	-	-
flowPB, %	4 (1, 17), (0)	4 (1, 22), (13)	-
flowPB ≥ 7% **	73 (40)	-	-
Blastoid histology	18 (10), (0)	8 (7), (0)	-
Bone Marrow Involved	95 (51), (0)	86 (75), (0)	-
dn involvement	184 (100), (0)	112 (98), (0)	-
EN involvement	95 (51), (0)	53 (46), (0)	-
*MIPI-standard*			
Low	110 (60)	70 (70)	
Intermediate	53 (29)	20 (17)	
High	22 (12)	25 (22)	
MV	0	-	
*MIPI-biologic*			
Low	49 (27)	25 (29)	
Intermediate	87 (47)	41 (48)	
High	49 (27)	20 (23)	
MV	0	29 (25)	
*MIPI-c*			
Low	91 (49)	42 (49)	
Low-Intermediate	49 (27)	30 (35)	
High-Intermediate	28 (15)	7 (8)	
High	17 (9)	7 (9)	
MV	0	29 (25)	

Values are the number (%) or median (interquartile range), as indicated, and the number of missing values (MV). * For the feature Age, categorization was done according to a cut-off of 60 years using a logistic regression model on the PFS. ** For the feature flowPB, categorization was done according to a cut-off of 7% using a logistic regression model on the PFS. Abbreviations. BMI: body mass index; ECOGps: Eastern Cooperative Oncology Group performance status; Sym: B symptoms; LDH: lactate dehydrogenase; ANC: absolute neutrophils count; Hb: hemoglobin level; ALT: alanine transferase; AST: aspartate aminotransferase; GGT: enzyme γ-glutamyl transferase level; ALP: alkaline phosphatase level; Ki-67: cell proliferation marker; flowPB: lymphoma involvement, measured with flow-cytometry on peripheral blood; dn: nodal involvement measured with CT scan; EN: extra-nodal involvement measured with CT scan; MIPI: mantle cell international prognostic index. PFS: progression free survival.

**Table 2:** Manual reduction of rules to obtain the smallest set that could correctly classify all the patients.

Risk	Formula	Criteria
Low	1	Normal L and A symptoms, normal albumin, Low Ki-67, Low LDH, and normal PLTs
High	2	Abnormal albumin
High	3	Normal albumin, high Ki-67, and normal PLTs
High	4	Normal albumin, high Ki-67, presence of BMInf and B Sym
High	5	Normal albumin, high Ki-67, presence of BMInf, normal Lymphocytes, and abnormal PLTs
High	6	Normal albumin, Low Ki-67, presence of BMInf and B Sym, and elevated LDH
High	7	Normal albumin, Low Ki-67, presence of BMInf and B Sym, Low LDH, normal Lymphocytes, and normal PLTs
Int	8	Neither Low or High

Abbreviations. Sym: symptoms; LDH: lactate dehydrogenase; PLTs: platelets; BMInf: tumor infiltration assessed with immunohistochemistry on a bone marrow biopsy; Int: intermediate risk.

**Table 3:** Comparisons between the eMIPI distribution and the MIPI-st and MIPI-b distributions in the training data set.

			MIPI-st			MIPI-b		
	Risk		Low	Int	High	Low	Int	High
		TOT (%)	110 (59)	53 (29)	32 (17)	49 (26)	87 (62)	49 (10)
eMIPI	Low	57 (31)	49	8	0	28	29	0
	Int	56 (30)	34	16	6	17	32	7
	High	72 (39)	27	29	16	4	26	42

Abbreviations. MIPI: mantle cell international prognostic index; MIPI-st: MIPI-standard; MIPI-b: MIPI biologic; Int: intermediate; TOT: total; eMIPI: engineered MIPI.

## Data Availability

The data presented in this study are available on request from the corresponding author.

## Supplementary Materials

The following are available online at https://www.mdpi.com/article/10.3390/cancers14010188/s1. Figure S1: Pipeline for data pre-processing, Figure S2: Flow diagram for preparation and validation of e-MIPI score, Figure S3: OS probability of patients included vs. patients excluded from training-set, Figure S4: Multicollinear analysis according to Spearman, Figure S5: Recursive feature extraction, Figure S6: Validation Series: MCL Younger, Figure S7: Validation Series: MCL Younger and Elderly, Table S1: Bivariate analysis, Table S2: Patients’ profiles, Table S3: Patients’ characteristics from the external validation series; Table S4: Power estimation in the validation cohort: validation series according to each cohort, Table S5: Descriptive statistics in the validation cohort: MCL Younger series, Table S6: Descriptive statistics in the validation cohort: MCL Younger and Elderly series, Supplementary Methods: Data Preparation. Data pre-processing: clustering analysis and feature reduction. Validation, Supplementary Results: Feature reduction. Validation.
