# Supplementary material for: A Clinical Prognostic Model Based on Machine Learning from the Fondazione Italiana Linfomi (FIL) MCL0208 Phase III Trial
Source: Cancers (Basel). 2021 Dec 31;14(1):188. doi: 10.3390/cancers14010188 (PMC8750124; doi:10.3390/cancers14010188)
Supplement: Supplementary file 1 [file cancers-14-00188-s001.zip › cancers-1462885-supplementary.pdf]

A clinical prognostic model based on machine learning from the Fondazione Italiana Linfomi (FIL) MCL0208 Phase III Trial.

Supplementary appendix

- **Supplementary Methods.** Data Preparation. Data pre-processing: clustering analysis and feature reduction. Validation.
- **Supplementary Results.** Feature reduction. Validation.
- **Figure S1.** Pipeline for data pre-processing.
- **Figure S2.** Flow diagram for preparation and validation of e-MIPI score.
- **Figure S3.** OS probability of patients included vs. patients excluded from training-set.
- **Table S1.** Bivariate analysis.
- **Figure S4.** Multicollinear analysis according to Spearman.
- **Figure S5.** Recursive feature extraction.
- **Table S2.** Patients' profiles.
- **Table S3.** Patients' characteristics from the external validation series.
- **Figure S6.** Validation Series: MCL Younger.
- **Figure S7.** Validation Series: MCL Younger and Elderly.
- **Table S4.** Power estimation: validation series according to each cohort.
- **Table S5.** Descriptive statistics in the validation cohort: MCL Younger.
- **Table S6.** Descriptive statistics in the validation cohort: MCL Younger and Elderly.
- **References.**

Supplementary Methods.

Data preparation

For the present analysis, we started from 34 clinical relevant baseline features available from eCRFs and laboratory data sources (Figure S1): sex, age at diagnosis, body mass index (BMI), eastern cooperative oncology group performance status (ECOGps), B symptoms, bulky disease higher 5 cm than not, lactate dehydrogenase (LDH) >/≤ upper limit of normal (ULN), platelets (PLTs), white blood cells count (WBC), absolute neutrophils count (ANC), lymphocytes (L) level, hemoglobin (Hb) level, alanine transferase (ALT) level, aspartate transferase (AST) level, creatinine level, total proteins, albumins level, bilirubin level, enzyme γ-glutamyl (GGT) transferase level, alkaline phosphatase (ALP) level, β2microglobulins (β2m) level, immunoglobulins G (IgG), A (IgA) and M (IgM) levels, lymphoma involvement by flow-cytometry on bone marrow (flowBM) and peripheral blood (flowPB), tumor infiltration assessed by Immuno-histochemistry on bone marrow (BMInf), Ki-67 proliferation index, blastoid histology, SOX11 proliferation index, IgH Homology, supra and sub diaphragmatic nodal involvement detected by computerized tomography (dn), extra-nodal involvement detected by computerized tomography (en), nodal involvement detected by hypercaptation positrons emission tomography (PET). Among these, 23 features were continuous (age, BMI, PLTs, WBC, ANC, L, Hb, ALT, AST, creatinine, total proteins, albumins, bilirubin, GGT, ALP, β2m, IgG, IgA, IgM, flowBM, flowPB, Ki-67 and IgH homology) while 11 were categorical (sex, ECOGps, B symptoms, bulky disease, LDH>/≤ULN, blastoid histology, BMInf, SOX11, dn, en and PET).

Of note, 8 features (6 continuous and 2 categorical) were not eligible due to the high number (≥40) of missing values (MVs) and were then excluded (β2m, IgG, IgA, IgM, flowBM, IgHOmo, SOX11 and PET. On the other hand, features with <15 MVs were imputed by the median of observations (AST, ALT, creatinine level, L, ANC, PLTs and BMInf).

Of the remaining 26 variables, 17 continuous were dichotomized, to be compared to the 9 binary ones, among these:

- 14 features dichotomized assuming the abnormal vs normal range according to the literature (medscape.org): BMI, PLTs, WBC, L, ANC, Hb, ALT, AST, Creatinine, total proteins, albumins, bilirubin, GGT and ALP.

Clustering analysis and features reduction

To identify the most influencing variables in the clusters, we performed a univariate analysis between each feature and the abovementioned groups, with the Chi-square test or Kruskal test, as appropriate. Features with $p<0.05$ were considered eligible for the subsequent steps (Supplementary Figure S2-C2). Moreover, for continuous variables, we performed a multicollinear analysis, according to Spearman, to discard highly correlated features (Supplementary Figure S2-C3).

Validation

Patient selection.

FIL-MCL0208 included patients with advanced stage MCL <65 years and suitable for autologous stem cell transplantation[1]. Thus, the MCL Younger trial included a similar patient cohort[2]. Treatment in FIL-MCL-0208 was 3xR-CHOP followed by a consolidation with MTX/high-dose cytarabine, autologous stem cell transplantation, and lenalidomide maintenance or observation according to randomization. Thus, treatment intensity was comparable to the experimental R-CHOP/R-DHAP group in MCL Younger that included high-dose cytarabine before autologous stem cell transplantation (ASCT). No maintenance was given in MCL Younger. The MCL Younger cohort has been used for primary external validation. The minimally relevant hazard ration between adjacent risk groups with power 80% and 90% and significance level 5% (unadjusted because of three groups in accordance with closed testing procedure) have been estimated separately for OS and compared with the observed hazard ratios in the training cohort. In case eMIPI showed a significant log-rank-test over three groups for OS and hazard ratios between the risk groups significantly different from 1 (5% significance level), prognostic power was compared with MIPI, MIPI-b, and MIPI-c on the experimental group of MCL Younger and the pooled group of MCL Younger and MCL Elderly[3,4]. Patients without MCL have been excluded.

Statistical analysis

Patients excluded from the external validation due to missing values on eMIPI variables have been compared to those included in terms of clinical characteristics and outcome. Kaplan-Meier estimates according to MIPI, MIPI-b, MIPI-c, and eMIPI were described for OS. Hazard ratios between adjacent risk groups have been calculated along with 95% confidence intervals. As measure for prognostic separation, Harrell's concordance (C) index was estimated, excluding unevaluable pairs (two censored survival times or one censored survival time shorter than the uncensored). In addition, more sensitive tools were also assessed as -2*log-likelihood [-2LL], Akaike [AIC] and Bayesian [BIC] Information Criteria.

Supplementary Results.

Feature reduction.

We correlated each individual feature included in the clustering analysis with every group obtained after the clustering analysis: accordingly, features with a $p<0.05$ were thus selected for the following steps (Table S1).

Multicollinear analysis among continuous features has shown in Figure S4. Correlations higher than 0.4 have been shown: L vs WBC from Figure S4-A with 0.71 of correlation; ANC vs WBC from Figure S4-B with 0.70 of correlation; L vs flowPB from Figure S4-C with 0.62 of correlation; PLTs vs ANC from Figure S4-D with 0.53 of correlation; Protein vs Alb from Figure S4-E with 0.44 of correlation; Hb vs Alb from Figure S4-F with 0.54 of correlation. Therefore, the following correlated features were discharged: WBC, flowPB, ANC; Protein, Hb. Finally, 14 variables were considered for the feature reduction step, namely: Sym, Bulky, LDH>/≤ULN, BMInf as categorical and age, BMI, PLTs, Hb, L, AST, Protein, Alb, ALP, Ki-67 as continuous. Figure S5 showed the accuracy level according to each model tested in the recursive process.

Validation.

<u>Data transformation</u>

1229 patients were registered in MCL Elderly and MCL Younger. The eMIPI variables were documented in the following way:

- BM involvement, LDH/ULN, B symptoms fully documented.
- Ki-67 assessed in accordance with guideline in 563 patients.
- Lymphocyte count documented as % of WBC, missing in 5, WBC documented in all patients in M/l; Lymphocyte count were calculated from Lymphocyte % values and WBC values.
- Platelets were documented in all patients in M/l.
- Albumin was documented in g/dl and missing in 182 patients.

<u>Power estimation, descriptive statistics and prognostic value according to eMIPI.</u>

Power estimation according to the validation series are shown in Table S3. Moreover, descriptive statistics of each sub-cohort are detailed in Figure S6, Figure S7, Table S4, and Table S5.

Figure S1. Pipeline for data pre-processing.

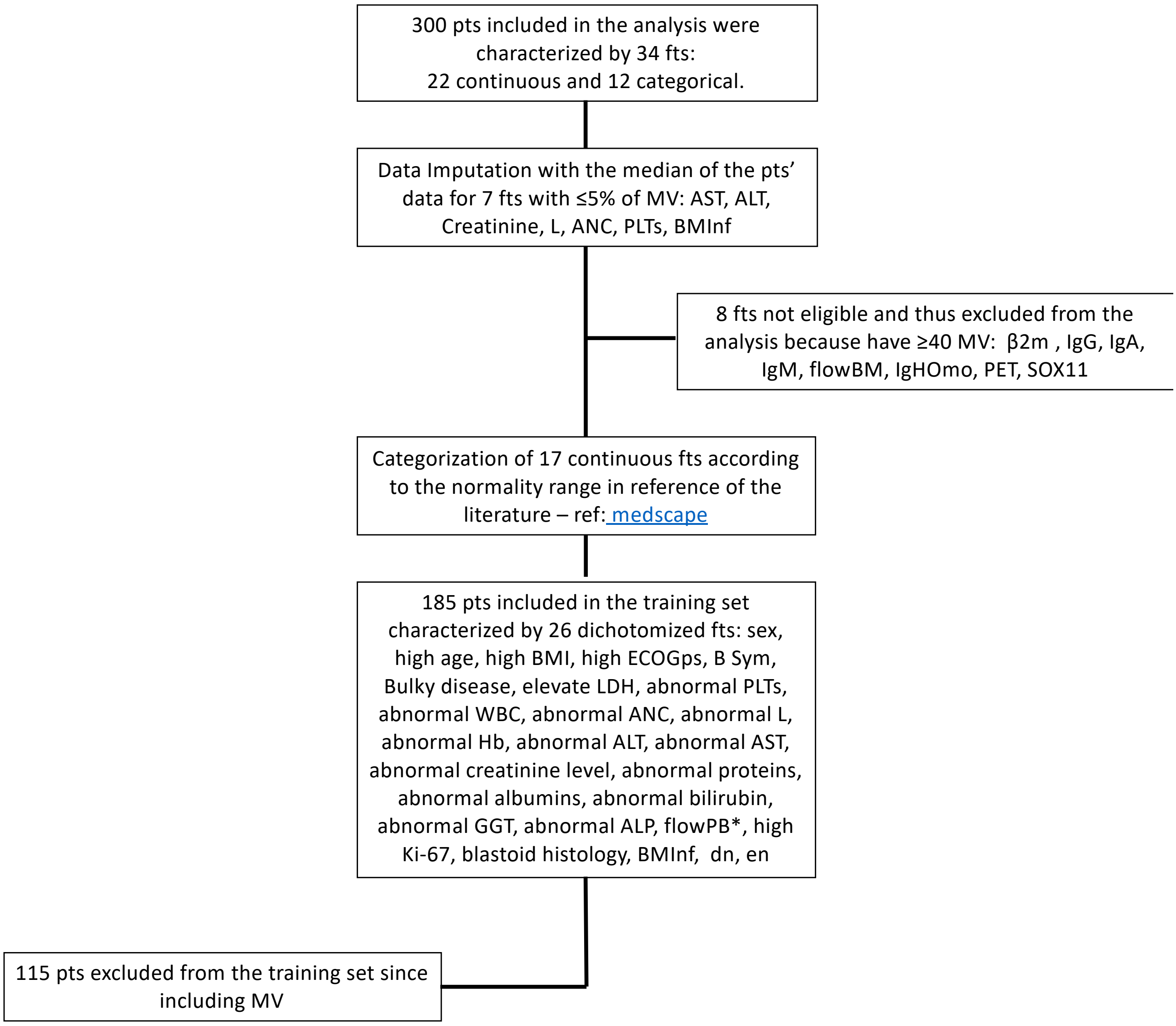

* *for the feature flowPB, categorization was done according to a cut-off of 7% using a logistic regression model on the PFS.*

Abbreviations. fts: features; pts: patients; eCRFs: electronic case report forms; BMI: body mass index; ECOGps: eastern cooperative oncology group performance status; Sym: B symptoms; LDH>/≤ULN: lactate dehydrogenase >/≤ upper level of normal; PLTs: platelets; WBC: white blood cells count; ANC: absolute neutrophils count; L: lymphocyte count; Hb: hemoglobin level; ALT: alanine transferase; AST: aspartate aminotransferase; Alb: albumin level; bili: bilirubin level. GGT: enzyme γ-glutamyl transferase level; ALP: alkaline phosphatase level; β2m: β2microglobulin level; IgG: g-immunoglobulins level; IgA: a-immunoglobulins level; IgM: m-immunoglobulins level; flowBM: lymphoma involvement by flow-cytometry on bone marrow; flowPB: lymphoma involvement by flow-cytometry on peripheral blood; BMInf: tumor infiltration assessed by immunohistochemistry on bone marrow biopsy; Hist: blastoid histology; IgHOmo: IgH omology; dn: nodal involvement by CT scan; en: extra-nodal involvement by CT scan; PET: hypercaptation by positrons emission tomography; MV: missing values. PFS: progression free survival.

Figure S2. Flow diagram for preparation and validation of e-MIPI score.

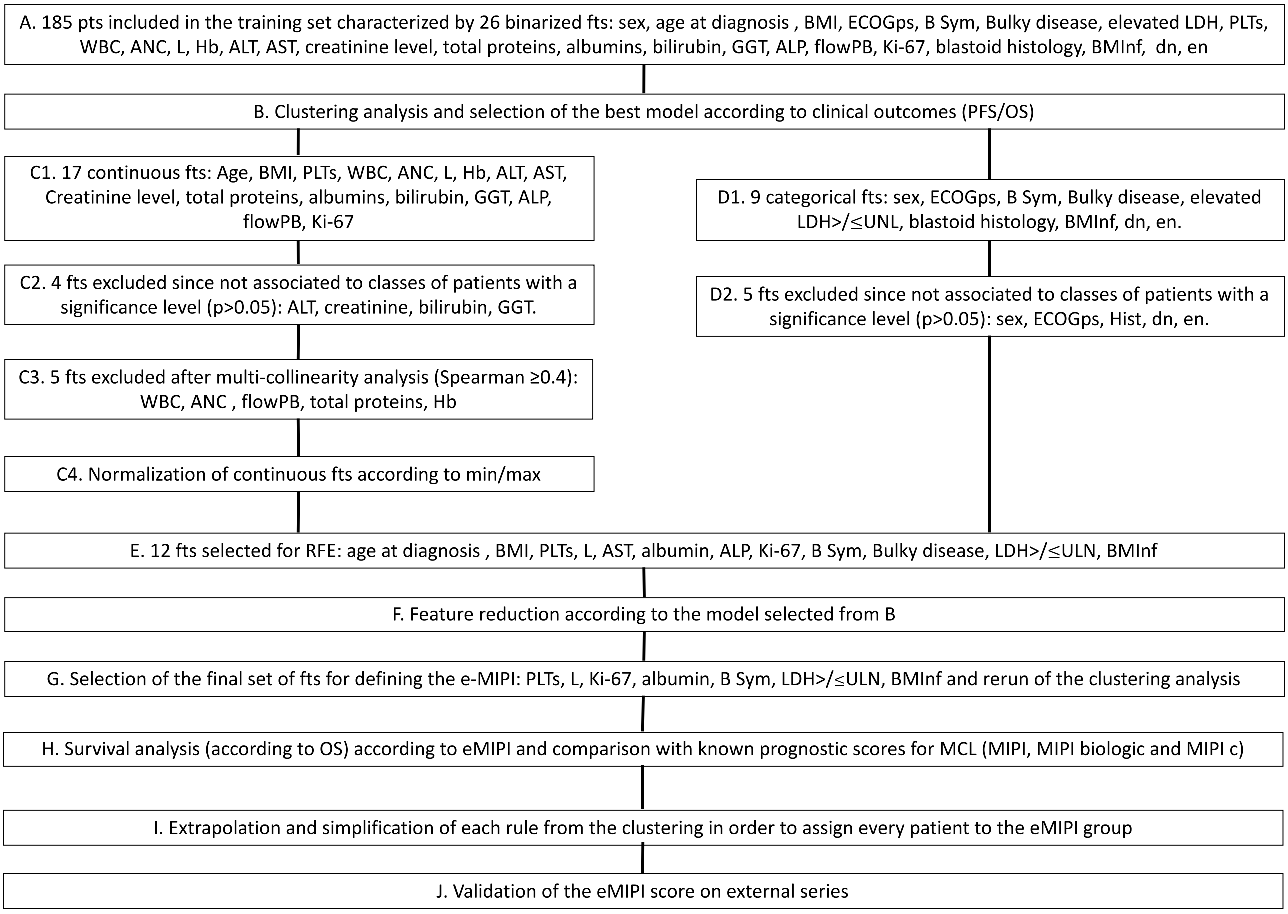

**variable with null variability.*

Abbreviations. eMIPI: engineered mantle cell lymphoma international prognostic index; fts: features; pts: patients; eCRFs: electronic case report forms; BMI: body mass index; ECOGps: eastern cooperative oncology group performance status; Sym: B symptoms; LDH>/≤ULN: lactate dehydrogenase >/≤ upper level of normal; PLTs: platelets; WBC: white blood cells count; ANC: absolute neutrophils count; L: lymphocyte count; Hb: hemoglobin level; ALT: alanine transferase; AST: aspartate aminotransferase; Alb: albumin level; bili: bilirubin level. GGT: enzyme γ-glutamyl transferase level; ALP: alkaline phosphatase level; β2m: β2microglobulin level; IgG: g-immunoglobulins level; IgA: a-immunoglobulins level; IgM: m-immunoglobulins level; flowBM: lymphoma involvement by flow-cytometry on bone marrow; flowPB: lymphoma involvement by flow-cytometry on peripheral blood; BMInf: tumor infiltration assessed by immunohistochemistry on bone marrow biopsy; Hist: blastoid histology; IgHOmo: IgH omology; dn: nodal involvement by CT scan; en: extra-nodal involvement by CT scan; PET: hypercaptation by positrons emission tomography; MV: missing values.PFS: progression free survival; OS: overall survival; MCL: mantle cell lymphoma, MIPI: MCL International prognostic Index; MIPI bio: MIPI biological.

Figure S3. OS probability of patients included vs. patients excluded from training-set.

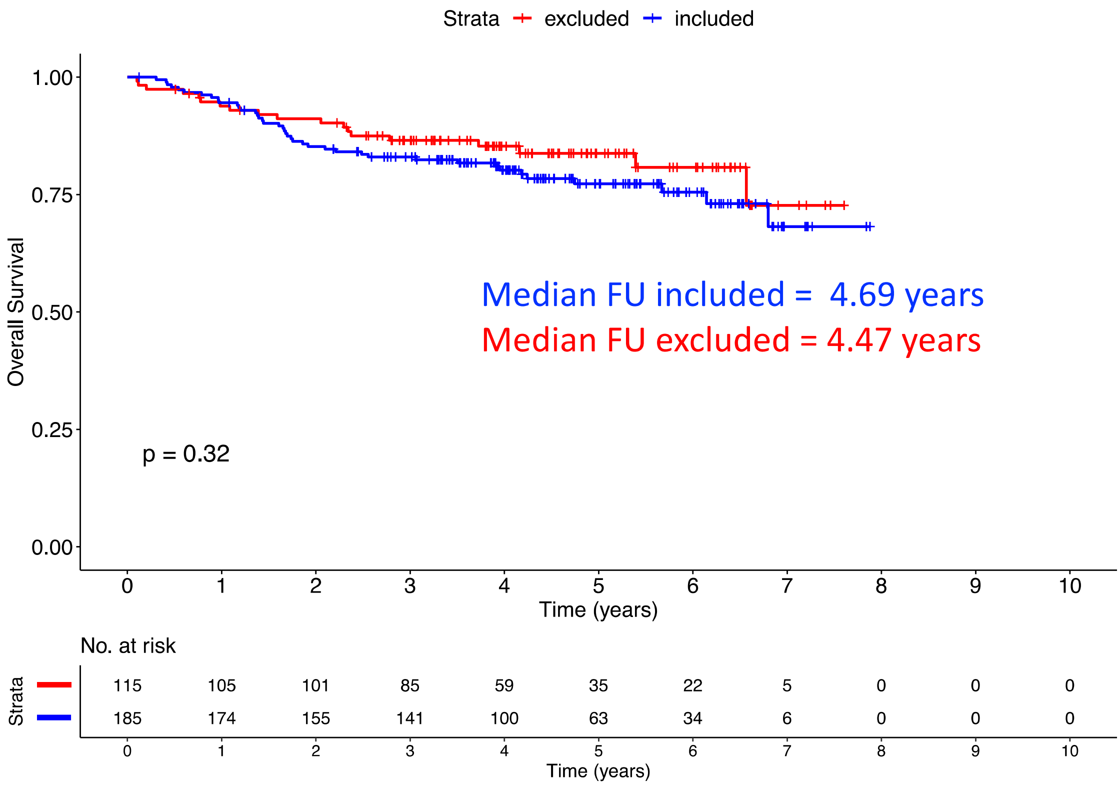

Abbreviations. FU: follow-up; No: number.

Table S1. Univariate analysis.

		Univariate Analysis
		p value
categorical	sex	0.20
	ECOGps	0.06
	B Sym	<0.01
	Bulky	<0.01
	LDH>/≤ULN	<0.01
	Hist	0.07
	BMInf	<0.01
	dn*	1
	en	0.28
continuous	Age	<0.01
	BMI	0.02
	PLTs	<0.01
	WBC	<0.01
	L	<0.01
	ANC	<0.01
	Hb	<0.01
	ALT	0.70
	AST	0.02
	creatinine	0.40
	total proteins	0.02
	albumin	<0.01
	bilirubin	0.10
	GGT	0.10
	ALP	0.01
	flowPB	<0.01
	Ki-67	<0.01

**variables with no variability, in fact both Chi-square and Kruskal Wallis test failed.*

Abbreviations. eMIPI: engineered mantle cell lymphoma international prognostic index; fts: features; pts: patients; eCRFs: electronic case report forms; BMI: body mass index; ECOGps: eastern cooperative oncology group performance status; Sym: B symptoms; LDHULN: lactate dehydrogenase >/≤ upper level of normal; PLTs: platelets; WBC: white blood cells count; ANC: absolute neutrophils count; L: lymphocyte count; Hb: hemoglobin level; ALT: alanine transferase; AST: aspartate aminotransferase; GGT: enzyme γ-glutamyl transferase level; ALP: alkaline phosphatase level; β2m: β2microglobulin level; IgG: g-immunoglobulins level; IgA: a-immunoglobulins level; IgM: m-immunoglobulins level;

flowBM: lymphoma involvement by flow-cytometry on bone marrow; flowPB: lymphoma involvement by flow-cytometry on peripheral blood; BMInf: tumor infiltration assessed by immunohistochemistry on bone marrow biopsy; Hist: blastoid histology; IgHOmo: IgH omology; dn: nodal involvement by CT scan; en: extra-nodal involvement by CT scan; PET: hyper-captation by positrons emission tomography.

Figure S4. multicollinear analysis according to Spearman.

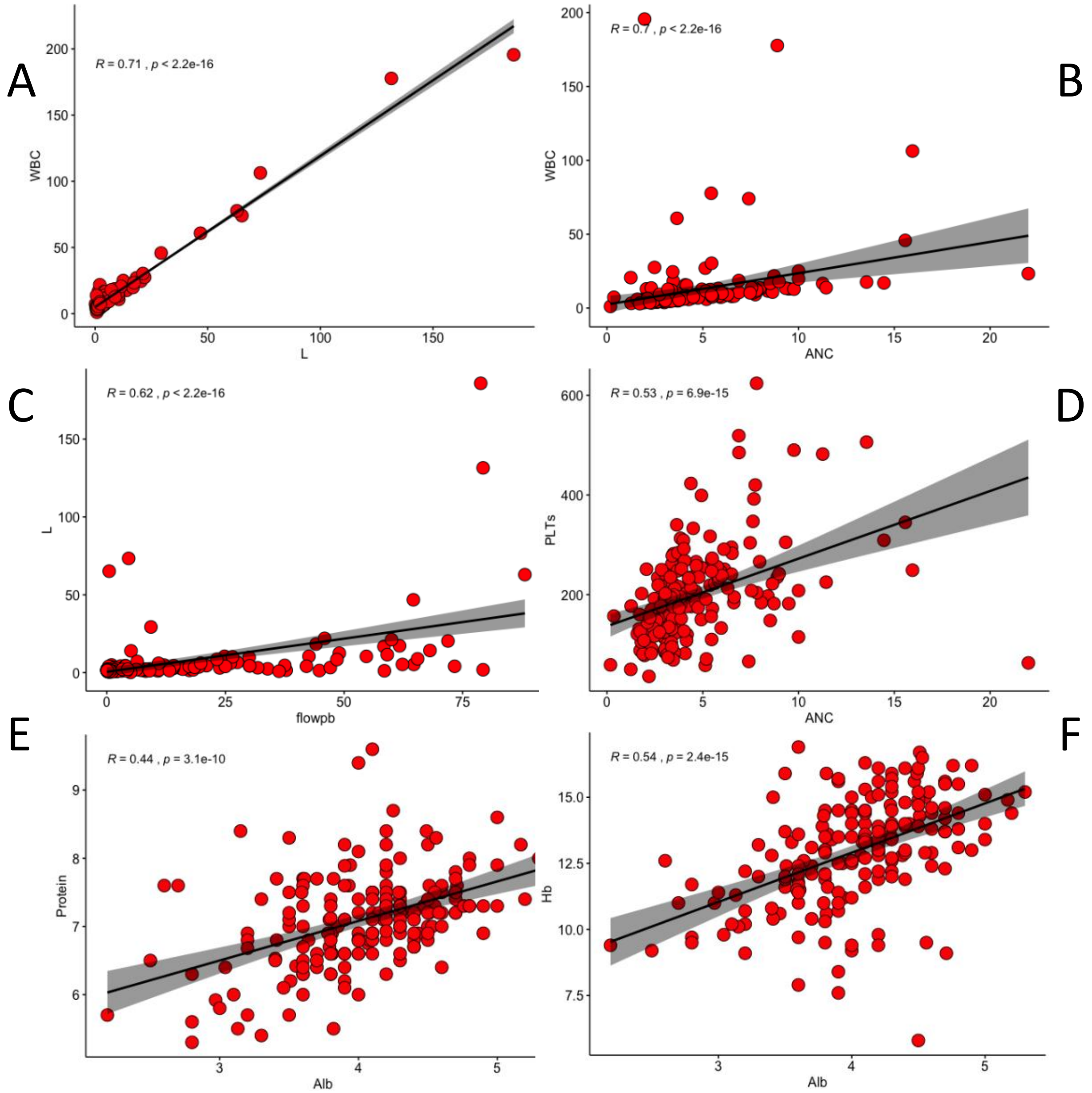

Each point is represented by an observed patient. The continuous line represents the linear regression of the whole of the points and the gray area is limited by the standard deviation calculated according to the correlation coefficient. Correlations higher than 0.4 have been shown: L vs WBC from Figure (A) with 0.71 of correlation; ANC vs WBC from Figure (B) with 0.70 of correlation; L vs flowPB from Figure (C) with 0.62 of correlation; PLTs vs ANC from Figure (D) with 0.53 of correlation; Protein vs Alb from Figure (E) with 0.44 of correlation; Hb vs Alb from Figure (F) with 0.54 of correlation.

Abbreviations. L: lymphocytes level count; WBC: white blood cells count; ANC: absolute neutrophils count; flowPB: lymphoma involvement by flow-cytometry on peripheral blood; PLTs: platelets level; Alb: albumin level; Hb: hemoglobin level. Sym: B symptoms.

Figure S5. Recursive feature extraction.

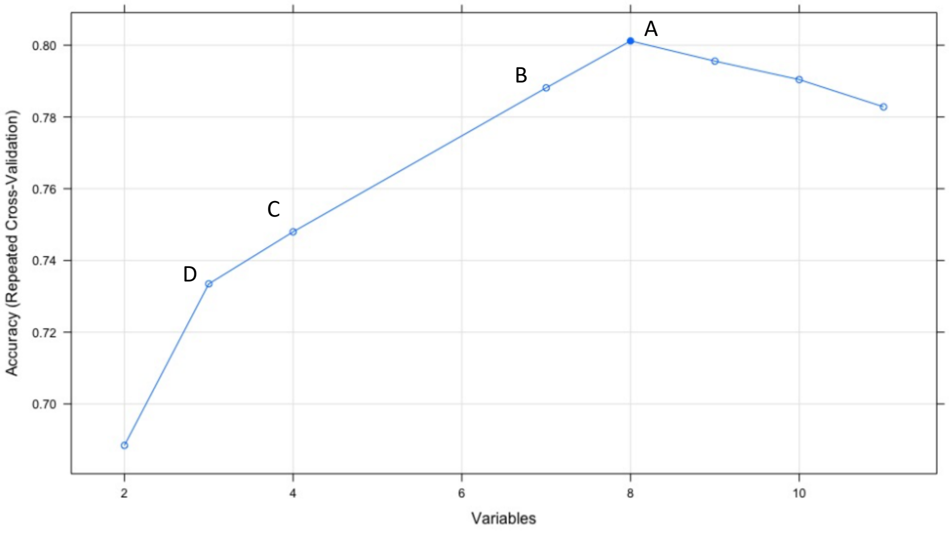

The RFE algorithm showed an increasing accuracy from a model with 8 variables (A, 80%) to a model with 7 (B, 76%), 4 (C, 75%) or 3 variables (D, 53%). Considering the minimal difference between A and B, the final model included 7 features for clinical applicability.

Abbreviations. RFE: recursive feature extraction.

Table S2. Patients' profiles.

		L	Sym	albumin	Ki-67	LDHULN	PLTs	BMInf
Low	Pt Profile 1	≥1 and ≤5	A	≥3.4 and ≤5.4	<30	≤1	≥150 and ≤450	No
Low	Pt Profile 2	≥1 and ≤5	A	≥3.4 and ≤5.4	<30	≤1	≥150 and ≤450	Yes
Int	Pt Profile 3	≥1 and ≤5	A	≥3.4 and ≤5.4	<30	≤1	<150 or >450	Yes
Int	Pt Profile 4	≥1 and ≤5	A	≥3.4 and ≤5.4	<30	≤1	<150 or >450	No
Int	Pt Profile 5	≥1 and ≤5	B	≥3.4 and ≤5.4	<30	≤1	<150 or >450	No
Int	Pt Profile 6	≥1 and ≤5	B	≥3.4 and ≤5.4	>=30	≤1	<150 or >450	No
Int	Pt Profile 7	≥1 and ≤5	B	≥3.4 and ≤5.4	<30	≤1	<150 or >450	Yes
Int	Pt Profile 8	<1 or >5	B	≥3.4 and ≤5.4	<30	≤1	<150 or >450	Yes
Int	Pt Profile 9	<1 or >5	B	≥3.4 and ≤5.4	<30	≤1	≥150 and ≤450	Yes
Int	Pt Profile 10	<1 or >5	A	≥3.4 and ≤5.4	<30	≤1	≥150 and ≤450	Yes
Int	Pt Profile 11	<1 or >5	A	≥3.4 and ≤5.4	<30	≤1	<150 or >450	Yes
Int	Pt Profile 12	<1 or >5	A	≥3.4 and ≤5.4	≥30	>1	<150 or >450	Yes
Int	Pt Profile 13	<1 or >5	A	≥3.4 and ≤5.4	<30	>1	<150 or >450	Yes
Int	Pt Profile 14	<1 or >5	A	≥3.4 and ≤5.4	<30	>1	≥150 and ≤450	Yes
Int	Pt Profile 15	<1 or >5	A	≥3.4 and ≤5.4	<30	>1	≥150 and ≤450	No
Int	Pt Profile 16	≥1 and ≤5	A	≥3.4 and ≤5.4	<30	>1	≥150 and ≤450	No
Int	Pt Profile 17	≥1 and ≤5	A	≥3.4 and ≤5.4	<30	>1	≥150 and ≤450	Yes
Int	Pt Profile 18	≥1 and ≤5	A	≥3.4 and ≤5.4	<30	>1	<150 or >450	Yes
High	Pt Profile 19	≥1 and ≤5	B	≥3.4 and ≤5.4	<30	>1	<150 or >450	Yes

High	Pt Profile 20	<1 or >5	B	≥3.4 and ≤5.4	<30	>1	<150 or >450	Yes
High	Pt Profile 21	<1 or >5	B	≤3.4 and ≤5.4	<30	>1	≥150 and ≤450	Yes
High	Pt Profile 22	<1 or >5	B	<3.4 and >5.4	<30	>1	<150 or >450	Yes
High	Pt Profile 23	≥1 and ≤5	B	<3.4 and >5.4	<30	>1	<150 or >450	Yes
High	Pt Profile 24	≥1 and ≤5	B	<3.4 and >5.4	<30	>1	≥150 and ≤450	Yes
High	Pt Profile 25	≥1 and ≤5	B	≥3.4 and ≤5.4	<30	>1	≥150 and ≤450	Yes
High	Pt Profile 26	≥1 and ≤5	B	≥3.4 and ≤5.4	≥30	>1	≥150 and ≤450	Yes
High	Pt Profile 27	≥1 and ≤5	B	≥3.4 and ≤5.4	≥30	≤1	≥150 and ≤450	Yes
High	Pt Profile 28	≥1 and ≤5	B	≥3.4 and ≤5.4	<30	≤1	≥150 and ≤450	Yes
High	Pt Profile 29	≥1 and ≤5	B	<3.4 and >5.4	<30	≤1	≥150 and ≤450	No
High	Pt Profile 30	≥1 and ≤5	B	<3.4 and >5.4	≥30	≤1	≥150 and ≤450	No
High	Pt Profile 31	≥1 and ≤5	B	<3.4 and >5.4	≥30	≤1	≥150 and ≤450	Yes
High	Pt Profile 32	<1 and >5	B	<3.4 and >5.4	≥30	≤1	≥150 and ≤450	Yes
High	Pt Profile 33	<1 and >5	B	<3.4 and >5.4	<30	≤1	≥150 and ≤450	Yes
High	Pt Profile 34	<1 and >5	A	<3.4 and >5.4	<30	≤1	≥150 and ≤450	Yes
High	Pt Profile 35	≥1 and ≤5	A	<3.4 and >5.4	≥30	≤1	≥150 and ≤450	Yes
High	Pt Profile 36	≥1 and ≤5	A	<3.4 and >5.4	≥30	≤1	<150 or >450	Yes
High	Pt Profile 37	≥1 and ≤5	A	<3.4 and >5.4	≥30	>1	<150 or >450	Yes
High	Pt Profile 38	≥1 and ≤5	B	<3.4 and >5.4	≥30	>1	<150 or >450	Yes
High	Pt Profile 39	≥1 and ≤5	B	<3.4 and >5.4	≥30	>1	≥150 and ≤450	Yes
High	Pt Profile 40	≥1 and ≤5	A	<3.4 and >5.4	≥30	>1	≥150 and ≤450	Yes
High	Pt Profile 41	<1 or >5	B	<3.4 and >5.4	≥30	>1	≥150 and ≤450	Yes

High	Pt Profile 42	<1 or >5	B	≥3.4 and ≤5.4	≥30	>1	≥150 and ≤450	No
High	Pt Profile 43	<1 or >5	B	≥3.4 and ≤5.4	≥30	>1	<150 or >450	Yes
High	Pt Profile 44	<1 or >5	B	≥3.4 and ≤5.4	≥30	≤1	<150 or >450	Yes
High	Pt Profile 45	<1 or >5	B	≥3.4 and ≤5.4	≥30	≤1	≥150 and ≤450	Yes
High	Pt Profile 46	<1 or >5	A	≥3.4 and ≤5.4	≥30	≤1	≥150 and ≤450	Yes
High	Pt Profile 47	<1 or >5	A	≥3.4 and ≤5.4	≥30	>1	≥150 and ≤450	Yes
High	Pt Profile 48	≥1 and ≤5	A	≥3.4 and ≤5.4	≥30	>1	≥150 and ≤450	Yes
High	Pt Profile 49	≥1 and ≤5	A	≥3.4 and ≤5.4	≥30	>1	<150 or >450	Yes
High	Pt Profile 50	≥1 and ≤5	A	≥3.4 and ≤5.4	≥30	≤1	<150 or >450	Yes
High	Pt Profile 51	≥1 and ≤5	A	≥3.4 and ≤5.4	≥30	≤1	≥150 and ≤450	Yes
High	Pt Profile 52	≥1 and ≤5	A	≥3.4 and ≤5.4	≥30	≤1	≥150 and ≤450	No
High	Pt Profile 53	<1 or >5	B	≥3.4 and ≤5.4	≥30	>1	≥150 and ≤450	No
High	Pt Profile 54	≥1 and ≤5	A	≥3.4 and ≤5.4	≥30	>1	≥150 and ≤450	No
High	Pt Profile 55	<1 or >5	B	≥3.4 and ≤5.4	≥30	>1	≥150 and ≤450	Yes

Abbreviations. Pts: patients; MCL: mantle cell lymphoma; MIPI: MCL international MCL prognostic index; eMIPI: electronic MIPI; L: lymphocytes; Sym: B symptoms; LDH>/≤ULN: lactate dehydrogenase >/≤ limit of normal; PLTs: platelets; BMInf: tumor infiltration assessed by immunohistochemistry on bone marrow biopsy.

Table S3. Patients' characteristics from the external validation series.

Variable	Value	Selected N	Median/ %	Range/ Freq	Excluded N	Median/ %	Range/ Freq	p-value
Age	years	254	54	30-65	359	56	33-66	0.0047
Age	>=60 years	254	24	60	359	31	110	0.067
Sex	male	254	80	204	359	80	286	0.92
ECOG PS	2	254	4	10	359	3	11	0.65
LDH	elevated	254	39	98	359	35	124	0.31
B-symptoms	present	254	37	94	359	36	130	0.86
Stage	1	254	0	1	359	0	1	0.47
	2	254	4	10	359	4	15	
	3	254	15	38	359	11	39	
	4	254	81	205	359	85	304	
Bone marrow	involved	254	75	190	359	76	273	0.77
Extranodal sites	>1	254	81	207	359	88	315	0.012
WBC	G/L	254	7.2	2.0-1105	359	7.7	1.1-805	0.33
Lymphocytes	G/L	254	1.8	0-530	359	2.0	0-318	0.053
Platelets	G/L	254	195	4.7-704	359	190	14-903	0.56
Hemoglobin	g/dL	254	13.3	4.2-19.1	359	13.1	4.8-16.9	0.29
Albumin	g/dL	254	4.2	1.9-7.2	259	4.2	2.4-7.1	>0.99
Ki-67	%	254	20	2-97	48	21	5-93	0.60
Ki-67 index	>=30%	254	28	70	48	27	13	>0.99
MCL cytology	blastic	209	11	24	150	4	6	0.012
MIPI score	1	254	5.51	4.21-8.68	359	5.62	4.07-7.98	0.0063
MIPI	Low	254	66	168	359	59	212	0.039
	Int		23	59		23	83	
	High		11	27		18	64	
MIPI-b	Low	254	30	76	48	23	11	0.43
	Int		46	116		56	27	
	High		24	62		21	10	
MIPI-c	Low	254	53	134	48	58	28	0.85
	Low-Int		28	72		29	14	
	High-Int		13	33		8	4	
	High		6	15		4	2	
eMIPI7	Low	254	22	57				
	Int		30	77				
	High		47	120				

Figure S6. Validation Series: MCL Younger.

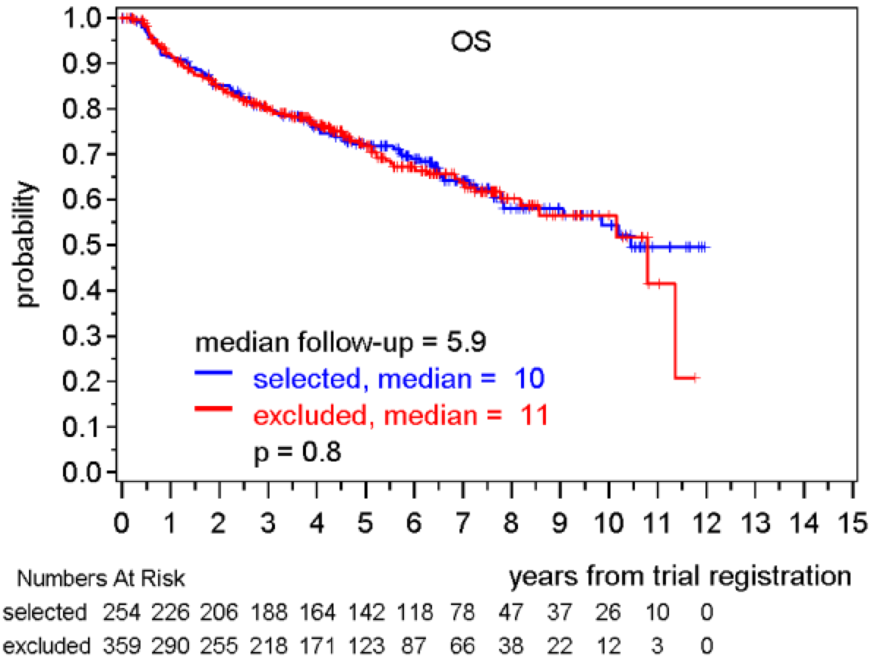

Abbreviations. OS: overall survival.

Figure S7. Validation Series: MCL Younger and Elderly.

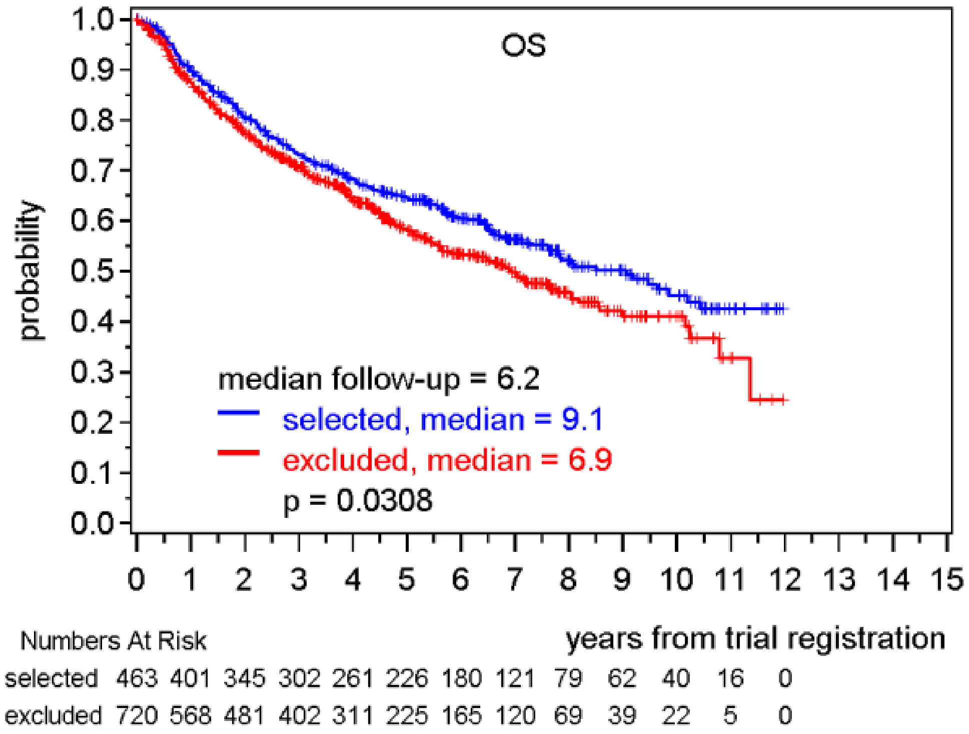

Abbreviations. OS: overall survival.

Table S4. Power estimation in the validation cohort: validation series according to each cohort.

Cohort	eMIPI group comparison	Outcome	No. of events	% higher group	Detectable Hazard ratio (alpha=5%, Power=90%)
**MCL Younger**	Intermediate versus low	OS	32	77/134	3.19
	High versus intermediate	OS	79	120/197	2.11
**MCL Younger & Elderly**	Intermediate versus low	OS	63	141/227	2.32
	High versus intermediate	OS	167	236/377	1.68

For OS, the MCL Younger cohort with pooled treatment groups yields numbers of events sufficient to detect the hazard ratios observed in the training cohort.

Abbreviations. No: number; MCL: mantle cell lymphoma; OS: overall survival.

Table S5. Descriptive statistics in the validation cohort: MCL Younger series

Variable	Value	Selected N	Median/ %	Range/ Freq	Excluded N	Median/ %	Range/ Freq	p-value
**Age at diagnosis**	years	254	54	30-65	359	56	33-66	0.0047
**MIPI score**	1	254	5.51	4.21-8.68	359	5.62	4.07-7.98	0.0063
**MIPI**	Low	254	66	168	359	59	212	0.039
	Int		23	59		23	83	
	High		11	27		18	64	
**MIPI-b**	Low	254	30	76	48	23	11	0.43
	Int		46	116		56	27	
	High		24	62		21	10	
**MIPI-c**	Low	254	53	134	48	58	28	0.85
	Low-Int		28	72		29	14	
	High-Int		13	33		8	4	
	High		6	15		4	2	
**eMIPI**	Low	254	22	57				
	Int		30	77				
	High		47	120				

Abbreviations. N: number; Freq: frequency; MIPI: MCL: mantle cell lymphoma; MIPI: international MCL prognostic index; MIPI-b: MIPI-biologic, eMIPI: engineered MIPI; Int: intermediate; OS: overall survival.

Table S6. Descriptive statistics in the validation cohort: MCL Younger and Elderly series.

Variable	Value	Selected N	Median/ %	Range/ Freq	Excluded N	Median/ %	Range/ Freq	p-value
**Age at diagnosis**	years	463	62	30-83	720	65	33-87	0.0003
**MIPI score**	1	463	5.83	4.21-8.68	720	5.97	4.07-8.84	<0.0001
**MIPI**	Low	463	40	187	720	33	240	<0.0001
	Int		35	161		29	210	
	High		25	115		38	270	
**MIPI-b**	Low	463	16	76	85	13	11	0.32
	Int		45	208		54	46	
	High		39	179		33	28	
**MIPI-c**	Low	463	32	148	85	38	32	0.66
	Low-Int		33	154		34	29	
	High-Int		24	111		19	16	
	High		11	50		9	8	
**eMIPI**	Low	463	19	86				
	Int		30	141				
	High		51	236				

Abbreviations. N: number; Freq: frequency; MIPI: MCL: mantle cell lymphoma; MIPI: international MCL prognostic index; MIPI-b: MIPI-biologic, eMIPI: engineered MIPI; Int: intermediate; OS: overall survival.

References.

1. Ladetto, M.; Cortelazzo, S.; Ferrero, S.; Evangelista, A.; Mian, M.; Tavarozzi, R.; Zanni, M.; Cavallo, F.; Di Rocco, A.; Stefoni, V.; et al. Lenalidomide maintenance after autologous haematopoietic stem-cell transplantation in mantle cell lymphoma: results of a Fondazione Italiana Linfomi (FIL) multicentre, randomised, phase 3 trial. *Lancet Haematol.* **2021**, *8*, e34–e44, doi:10.1016/S2352-3026(20)30358-6.
2. Hermine, O.; Hoster, E.; Walewski, J.; Bosly, A.; Stilgenbauer, S.; Thieblemont, C.; Szymczyk, M.; Bouabdallah, R.; Kneba, M.; Hallek, M.; et al. Addition of high-dose cytarabine to immunochemotherapy before autologous stem-cell transplantation in patients aged 65 years or younger with mantle cell lymphoma (MCL Younger): a randomised, open-label, phase 3 trial of the European Mantle Cell Lymphoma N. *Lancet* **2016**, *388*, 565–575, doi:10.1016/S0140-6736(16)00739-X.
3. Kluin-Nelemans, H.C.; Hoster, E.; Hermine, O.; Walewski, J.; Geisler, C.H.; Trneny, M.; Stilgenbauer, S.; Kaiser, F.; Doorduijn, J.K.; Salles, G.; et al. Treatment of Older Patients With Mantle Cell Lymphoma (MCL): Long-Term Follow-Up of the Randomized European MCL Elderly Trial. *J. Clin. Oncol.* **2019**, *38*, 248–256, doi:10.1200/JCO.19.01294.
4. Kluin-Nelemans, H.C.; Hoster, E.; Hermine, O.; Walewski, J.; Trneny, M.; Geisler, C.H.; Stilgenbauer, S.; Thieblemont, C.; Vehling-Kaiser, U.; Doorduijn, J.K.; et al. Treatment of older patients with mantle-cell lymphoma. *N. Engl. J. Med.* **2012**, *367*, 520–531, doi:10.1056/NEJMoa1200920.
